# Abstract of Proceedings of the Buffalo Medical Association

**Published:** 1869-10

**Authors:** T. M. Johnson

**Affiliations:** Sec’y.


ART. II.—*Abstract of Proceedings of the Buffalo Medical Association :*

BUFFALO, Tuesday Evening, Oct., 5th, 1869.

The meeting was called to order by the President.

Members present,—Drs. MINER, DIEHL, ABBOTT, RING, WETMORE, ROCHESTER, TAYLOR and JOHNSON. Dr. F. W. ABBOTT, reported the following case:

Joseph Bachman, a mechanic, aged about 14 years, came to my office, Aug. 3d., and gave the following history of his case. One week before he was struck upon the left eye with an iron chip, which did not penetrate the ball, but fell to the floor. He immediately experienced an excruciating pain, with total loss of vision, which continued about an hour when the pain began to abate, and has not recurred with much severity. His vision was soon partially restored, but continued very dim, and he saw large black spots floating continually before his eye.

Upon examination I found considerable congestion of a section of the conjunctiva upon the temporal side, evidently traumatic in origin. The cornea was normal, the iris of natural color but strongly dilated, and completely immovable. Upon testing his vision with glasses, his accommodation was found to be completely paralyzed, and he had a hypermetropia of one-ninth, with vision of about two-thirds. I then examined him with the ophthalmoscope and found some hæmorrhagic effusion into the vitreous, which prevented a satisfactory view of the fundus.

Aug. 12th he came again. By this time the conjunctiva had resumed its normal appearance, the iris was still dilated, but responded slightly to the light, accommodation was partially restored, the hypermetropia was only 1-24th, and this being corrected his vision was 1. At one foot he could read No. 3 of Snellen. An ophthalmoscopic examination showed that the vitreous was clearer than it was nine days previous, and a perfect view of the fundus oculis was obtained. The optic disc was normal. Near its lower and nasal side, (inverted image,) was a white line about one and one-half D long and from one-fourth to one-third D broad, (D diameter of optic disc, the unit of measurement in ophthalmic observation,) which I recognized as a rupture of the choroid. There were no retinal vessels in this portion of the field.

The point of peculiar interest in this case is the sudden and transient development of so high a degree of hypermetropia, which is not mentioned as occurring in any of the cases of rupture of the choroid upon record so far as I can learn. In the archives of Ophthalmology and Otology, Vol. 1 No. 1., Prof. Knapp, publishes a report of 12 cases of choroidal rupture which were observed by himself and 17 cases which had been described by others, which he says comprises all the cases upon record except two, but, in none of these is there mention made of a transient hypermetropia. The other eye was perfectly immetropic so far as I could discover without paralyzing the accommodation. What was the cause of this short lived anomaly of refraction which we have been taught to consider the result solely of an anatomical defect, I am unable even to conjecture satisfactorily, and hope to receive some light upon it by bringing the case before this association. I regret, exceedingly my inability to follow out this case farther, but in spite of reiterated promises to come again and report progress, my patient never returned after the second visit.

Dr. MINER spoke of the interest and rarity of such injuries of the choroid tunic of the eye ball. They are now believed to occur quite frequently from blows and other similar accidents, but the true nature of the lesion is often overlooked. These cases are doubtless much more frequent than would appear from reports, since these intra-ocular changes could not be recognised at all, until the discovery of the Ophthalmoscope, and are now only observed in the comparatively rare instances where the cases fall under the observation of a physician familiar with the use of the Ophthalmoscope, and the appearances of the normal *fundus oculi.*

In order to arrive at any conclusions in regard to the causes of the hypermetropia observed and described by Dr. ABBOTT, it would be necessary to reflect a moment upon the necessary changes in the eye to produce this conditton. If the eye is hypermetropic, parallel rays of light are focused beyond the retina, and Dr. ABBOTT has shown how the form of the globe is generally in fault in this condition of long-sightedness or hypermetropia; but there are conditions of the refracting media which may also produce this—the lens may not be of natural power, or the cornea may be defective in refractive power; or, as has been already pointed out, the globe may be too short in its antero-posterior diameter.

The concussion of a blow can hardly be supposed to change the form of the globe, or at least to lessen the antero-posterior diameter of the sclerotic tunic of the eye-ball. A blow might rupture the vessels of the more vascular and delicate choroid, causing effusion of blood or serum between it and the sclerotic, thus crowding forward the retina and producing hypermetropia; and this might also be temporary, disappearing gradually as the effused products were absorbed. This explanation would be quite satisfactory theoretically; but, if effusion had thus taken place, the ophthalmoscopic inspection should have shown it.

Escape of the aqueous humor, and the consequent flattening of the cornea, would also produce similar effects upon the rays of light, so that they might not be focused soon enough, passing to a point beyond the retina; but no such escape was noticed. Changes in the lens, in its power or in its place, would influence the rays of light. The lens was certainly more or less affected, as it did not accommodate to near objects. Paralysis of the ciliary muscle had been effected by the injury, and thus accommodation destroyed. Perhaps the explanation of the transient hypermetropia might be found in the diminution of the refractive power of the lens. Taking the premises as given, he could not, at that moment, satisfactorily explain the phenomena, but thought that it must have consisted in some of the changes indicated. The case was a very interesting and instructive one, and he felt under great obligation to Dr. ABBOTT for report of it.

Dr. ROCHESTER wished to report a case which was of interest pathologically, and also illustrate the dangers to which enthusiastic persons may be led, especially when their enthusiasm exceeds their knowledge. A gentleman in this city, who has been engaged in different occupations, has a very fine laryngoscope which he delights to use, and is quite enthusiastic upon the subject of laryngoscopy. A patient who had been under his treatment, a man of forty-five ears of age, came to my office and told me that the enthusiastic gentlemen above mentioned had examined his throat, and said he had a large swelling or tumor in his larynx, which rose and fell with his breathing. At the man's request I made a careful examination of his throat, but could find nothing abnormal, and became satisfied that there was no disease of the larynx at all. I afterwards examined his thorax and found a clear aneurismal thrill in the region of the arch of the aorta. The patient said that he was not surprised at the information, for he had been told by Dr. ELERY SMITH, ten years ago, that he had heart disease. I called the attention of the laryngoscopist to the real condition of the case, but failed to convince him that the larynx was in healthy condition. A few days since the patient died; and I was present at the *post mortem*, which showed a healthy state of the larynx and trachea, but a large aneurism at the arch of the aorta. He died of angina pectoris in a fit of dyspnœa.

Dr. ROCHESTER, in speaking of prevailing diseases said that a peculiar kind of fever is said to be prevailing in the vicinity of Maple Street, commencing with intermittent symptoms, with severe pains in the back, and soon taking on typhoid symptoms and running a severe course, frequently fatal. Quinine does not seem to be beneficial. Adjourned.

T. M. JOHNSON, Sec'y.

